# Epidemiological aspects of and risk factors for wheezing in the first year of life*

**DOI:** 10.1590/S1806-37132014000600005

**Published:** 2014

**Authors:** Hamilton Rosendo Fogaça, Fernando Augusto de Lima Marson, Adyléia Aparecida Dalbo Contrera Toro, Dirceu Solé, José Dirceu Ribeiro

**Affiliations:** Fundação Universidade Regional de Blumenau, Department of Medicine, Blumenau, Brazil. Department of Medicine, Fundação Universidade Regional de Blumenau, Blumenau, Brazil; State University at Campinas, School of Medical Sciences, Departments of Medical Genetics and Pediatrics, Campinas, Brazil. Departments of Medical Genetics and Pediatrics, State University at Campinas School of Medical Sciences, Campinas, Brazil; State University at Campinas, School of Medical Sciences, Department of Pediatrics, Campinas, Brazil. Department of Pediatrics, State University at Campinas School of Medical Sciences, Campinas, Brazil; State University at Campinas, School of Medical Sciences, Department of Pediatrics, São Paulo, Brazil. Allergy, Clinical Immunology, and Rheumatology Section of the Department of Pediatrics, Federal University of São Paulo Paulista School of Medicine, São Paulo, Brazil; State University at Campinas, School of Medical Sciences, Center for Pediatric Research, Campinas, Brazil. Department of Pediatrics, Center for Pediatric Research, Pulmonary Physiology Laboratory, State University at Campinas School of Medical Sciences, Campinas, Brazil

**Keywords:** Asthma, Prevalence, Risk factors

## Abstract

**OBJECTIVE:**

To determine, in a sample of infants, the prevalence of and risk factors for occasional wheezing (OW) and recurrent wheezing-wheezy baby syndrome (WBS).

**METHODS:**

Parents of infants (12-15 months of age) completed the International Study of Wheezing in Infants questionnaire.

**RESULTS:**

We included 1,269 infants residing in the city of Blumenau, Brazil. Of those, 715 (56.34%) had a history of wheezing, which was more common among boys. The prevalences of OW and WBS were 27.03% (n = 343) and 29.31% (n = 372), respectively. On average, the first wheezing episode occurred at 5.55 ± 2.87 months of age. Among the 715 infants with a history of wheezing, the first episode occurred within the first six months of life in 479 (66.99%), and 372 (52.03%) had had three or more episodes. Factors associated with wheezing in general were pneumonia; oral corticosteroid use; a cold; attending daycare; having a parent with asthma or allergies; mother working outside the home; male gender; no breastfeeding; and mold. Factors associated with WBS were a cold; physician-diagnosed asthma; ER visits; corticosteroid use; pneumonia; bronchitis; dyspnea; attending daycare; bronchodilator use; having a parent with asthma; no breastfeeding; mother working outside the home; and a dog in the household.

**CONCLUSIONS:**

The prevalence of wheezing in the studied population was high (56.34%). The etiology was multifactorial, and the risk factors were intrinsic and extrinsic (respiratory tract infections, allergies, attending daycare, and early wheezing). The high prevalence and the intrinsic risk factors indicate the need and the opportunity for epidemiological and genetic studies in this population. In addition, mothers should be encouraged to prolong breastfeeding and to keep infants under six months of age out of daycare.

## Abstract (pt)

**OBJETIVO:**

Determinar a prevalência e os fatores de risco para sibilância ocasional (SO) e recorrente - síndrome do lactente sibilante (SLS).

**MÉTODOS:**

Pais de lactentes (12-15 meses de idade) responderam o questionário escrito *Estudio Internacional de Sibilancias en Lactantes*.

**RESULTADOS:**

Foram incluídos 1.269 lactentes residentes na cidade de Blumenau (SC). Desses, 715 (56,34%) apresentavam sibilância, sendo essa mais frequente nos meninos. As prevalências de SO e SLS foram de 27,03% (n = 343) e 29,31% (n = 372), respectivamente. O primeiro episódio de sibilância ocorreu aos 5,55 ± 2,87 meses de idade em média. Em 479/715 (66,99%) lactentes com sibilância, o primeiro episódio ocorreu nos primeiros seis meses de vida, enquanto 372/715 (52,03%) apresentaram três ou mais episódios. Os fatores associados com sibilância foram pneumonia; uso de corticoide oral; resfriado; frequência a creches; pais com asma e/ou alergia; mãe com emprego; gênero masculino; sem aleitamento materno; e mofo. Os fatores associados com SLS foram resfriado; diagnóstico médico de asma; visitas a serviço de emergência; uso de corticoide oral/inalatório; pneumonia, bronquite; dispneia; frequência a creches; uso de broncodilatador; pais com asma; sem aleitamento materno; mãe com emprego; e presença de cachorro na residência.

**CONCLUSÕES:**

A prevalência de sibilância na população estudada foi elevada, sendo multifatorial e com fatores de risco intrínsecos e extrínsecos (infecções do trato respiratório, alergia nos pais, frequência a creches e idade precoce de chiado). A elevada prevalência e os fatores de risco intrínsecos encontrados indicam a necessidade e a oportunidade para estudos epidemiológicos e genéticos nessa população. Além disso, estratégias para que as mães aumentem o período de amamentação ao seio e evitem que seus filhos frequentem creches antes dos seis primeiros meses de vida devem ser estimuladas.

## Introduction

Wheezing in the first year of life can be classified as occasional wheezing (OW) or recurrent wheezing, the latter being known as wheezy baby syndrome (WBS). Both are common clinical conditions that are heterogeneous and are caused by numerous diseases and airway injury, manifesting clinically and biochemically as a variety of phenotypes.^(^ 1 ^,^ 2 ^)^

Some children have transient early WBS, whereas others have early respiratory symptoms that can be the first manifestation of asthma. In recent decades, studies have investigated the risk factors for WBS and the relationship between WBS and the development of asthma, issues that are central to asthma prevention.^(^ 1 ^)^ Most school-age children with a history of asthma and impaired pulmonary function have a history of OW or WBS in the first year of life. 

Studies conducted in Brazil^(^ 2 ^-^ 8 ^)^ and other countries^(^ 9 ^-^ 12 ^)^ have shown that OW and WBS are common in the first year of life, their prevalence ranging from 13.0% to 80.3%. Although the prevalence of WBS is high, it has been reported that WBS disappears after early childhood.^(^ 1 ^)^ Because the prevalence of wheezing is high, it is necessary to determine the severity of and risk factors for OW and WBS in the first year of life in Brazil. 

OW and WBS in the first year of life are noteworthy for two reasons: (i) individuals exposed to risk factors for OW or WBS are more likely to develop asthma; (ii) most patients with WBS have viral exacerbations, without atopy, and do not develop asthma.^(^ 2 ^,^ 13 ^)^

Prevalence studies of OW and WBS in the first year of life have identified several risk factors, including a family history of asthma; certain dietary and occupational habits during pregnancy; passive smoking; lack of breastfeeding; male gender; attending daycare; certain environmental pollutants (cigarette smoke and sensitization to aeroallergens, including dust mite aeroallergens, cockroach aeroallergens, and animal dander); pneumonia or viral respiratory infections caused by respiratory syncytial virus or rhinovirus; and use of antibiotics or paracetamol.^(^ 2 ^-^ 12 ^)^

The *Estudio Internacional de Sibilancias en Lactantes* (EISL, International Study of Wheezing in Infants) questionnaire was created in 2005 in order to evaluate the prevalence, severity, and characteristics of wheezing in the first year of life in Latin America and Europe.^(^ 10 ^)^

The EISL questionnaire was used in an international multicenter study involving Latin American countries, Spain, and the Netherlands, having been standardized and validated for use in all of the aforementioned countries, including Brazil.^(^ 6 ^,^ 10 ^,^ 14 ^-^ 16 ^)^

The objective of the present study was to determine the prevalence of and risk factors for OW and WBS in the first year of life in a sample of infants in the city of Blumenau, Brazil. 

## Methods

A prospective cross-sectional study was conducted at 43 health care clinics in the city of Blumenau, the EISL questionnaire being used.^(^ 9 ^-^ 12 ^)^

In order to calculate the sample size, we used the method used in the EISL,^(^ 14 ^)^ considering that the prevalence of wheezing was 25-30%. Taking into account a power of 95% and an α of 0.01, we calculated that 1,100 infants were required. 

The EISL questionnaire consists of 50 questions regarding wheezing, risk factors, demographic characteristics, and environmental characteristics, having previously been translated into Brazilian Portuguese and validated for use in the Brazilian population.^(^ 2 ^)^

The EISL questionnaire was administered by the principal investigator and previously trained health care clinic staff and was completed by the caregivers of the infants (age, 12-15 months), in accordance with the method proposed and used in the original study,^(^ 14 ^)^ during visits for routine immunization or routine child care visits to the health care clinics over a period of 18 months. 

All of the infants whose legal guardians gave written informed consent were included in the present study. The study was approved by the Research Ethics Committee of the *Fundação Universidade Regional de Blumenau*, located in the city of Blumenau (Protocol no. 039/08). 

Infants who had previously been diagnosed with genetic disease, neuropathy, myopathy, heart disease, primary or secondary malnutrition, cystic fibrosis, or somatic malformations were excluded, as were those with limited life expectancy. The infants were divided into three groups: the WBS group, comprising infants who had had three or more episodes of wheezing; the OW group, comprising infants who had had fewer than three episodes of wheezing; and the nonwheezing group, comprising infants who had never wheezed. 

The data obtained by the EISL questionnaire were coded, entered into a Microsoft Excel^(r)^ 2007 database, and statistically analyzed with the use of the Statistical Package for the Social Sciences, version 18.0 (SPSS Inc., Chicago, IL, USA), for Windows. Bivariate and multivariate logistic regression analyses were used in order to compare risk factors between wheezing and nonwheezing infants, as well as between the OW and WBS groups. The results are presented as OR and 95% CI. The variables that showed significant values for the studied association were described. Values of α < 0.05 were considered statistically significant. 

## Results

Caregivers of 1,269 infants 12-15 months of age were interviewed. Of those infants, 1,211 (95.43%) were White. In addition, 715 (56.34%) had had episodes of wheezing. Of those 715 infants, 343 (27.03%) had OW and 372 (29.31%) had WBS. 

The EISL questionnaire was completed by mothers, fathers, and others in 1,073 (84.55%), 106 (8.35%), and 90 (7.09%), respectively. The age distribution was as follows: 12 months, in 549 (43.26%); 13 months, in 295 (23.25%); 14 months, in 331 (26.08%); and 15 months, in 94 (7.41%). 

**Figure 1 -:**
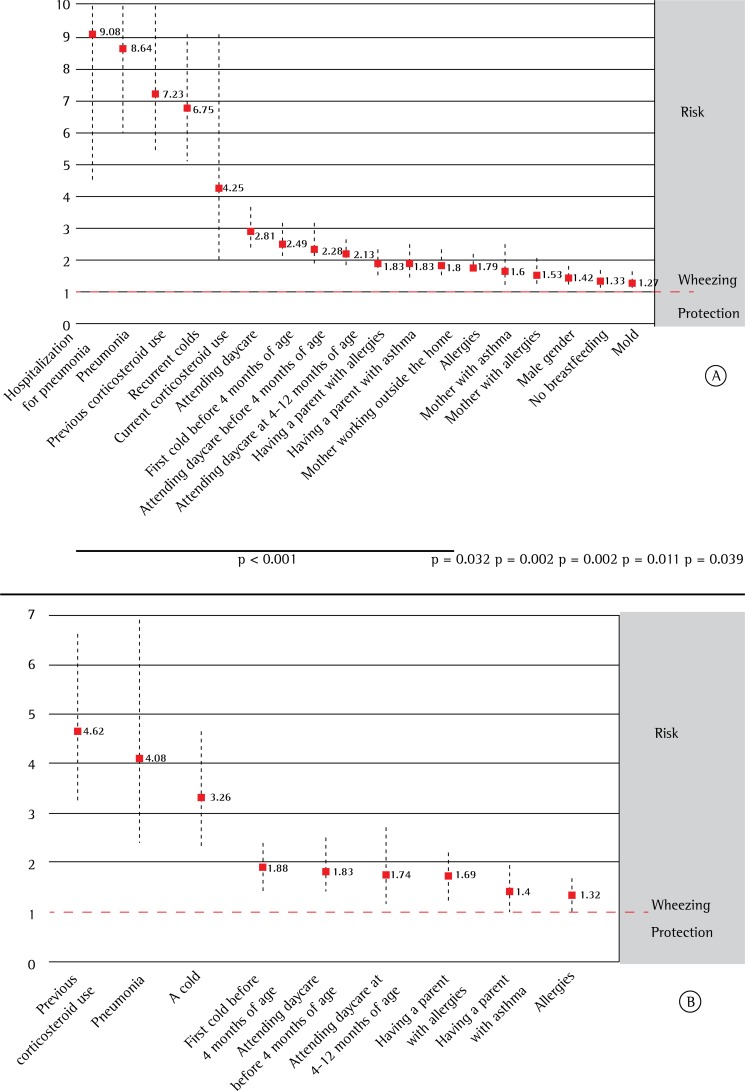
Factors associated with wheezing in the first 12 months of life (wheezing infants, n = 715; nonwheezing infants, n = 554; total, n = 1,269). In A, bivariate logistic regression analysis; in B, multivariate logistic regression analysis.

The risk factors identified and showing positive ORs are described in the figures and tables. Figure 1A and Table 1 show the bivariate analysis for the presence of wheezing in the study population. Figure 1B and Table 1 show the multivariate analysis for the presence of wheezing in the study population. Figures 1A/B and Table 1 show data for the sample as a whole, comparisons being made between wheezing infants (the OW and WBS groups taken together) and nonwheezing infants. Table 1 shows the distribution of risk factors for wheezing, together with their respective ORs and 95% CIs, among the infants studied. 

**Table 1 -:** Factors associated with wheezing in the first year of life (wheezing infants, n = 715; nonwheezing infants, n = 554; total, n = 1,269).

Type of analysis	Variable	OR	95% CI	p
Bivariate	Hospitalization for pneumonia	9.08	4.36-18.93	< 0.001
	Pneumonia	8.64	5.3-14.1	< 0.001
	History of oral corticosteroid use	7.23	5.16-10.13	< 0.001
	More than three colds in the first year	6.75	5.00-9.12	< 0.001
	Current oral corticosteroid use	4.25	1.98-9.14	< 0.001
	Attending daycare	2.81	2.30-3.64	< 0.001
	First cold before 4 months of age	2.49	1.98-3.15	< 0.001
	Attending daycare before 4 months of age	2.28	1.61-3.22	< 0.001
	Attending daycare after 4 months of age	2.13	1.67-2.71	< 0.001
	Having a parent with allergies	1.83	1.46-2.31	< 0.001
	Having a parent with asthma	1.83	1.39-2.40	< 0.001
	Mother working outside the home	1.8	1.42-2.27	< 0.001
	History of allergies	1.79	1.43-2.23	< 0.001
	Mother with asthma	1.6	1.03-2.49	0.0318
	Mother with allergies	1.53	1.16-2.02	0.0023
	Male gender	1.42	1.13-1.77	0.002
	No breastfeeding	1.33	1.06-1.66	0.0108
	Mold	1.27	1.01-1.60	0.0386
Multivariate	History of oral corticosteroid use	4.62	3.20-6.67	< 0.001
	Pneumonia	4.08	2.40-6.93	< 0.001
	More than three colds in the first year	3.26	2.31-4.60	< 0.001
	First cold before 4 months of age	1.88	1.42-2.48	< 0.001
	Attending daycare before 4 months of age	1.83	1.36-2.46	0.0001
	Attending daycare after 4 months of age	1.74	1.13-2.68	0.0118
	Having a parent with allergies	1.69	1.28-2.23	0.0002
	Having a parent with asthma	1.40	1.00-1.95	0.0496
	History of allergies	1.32	1.01-1.72	0.0442

**Figure 2 -:**
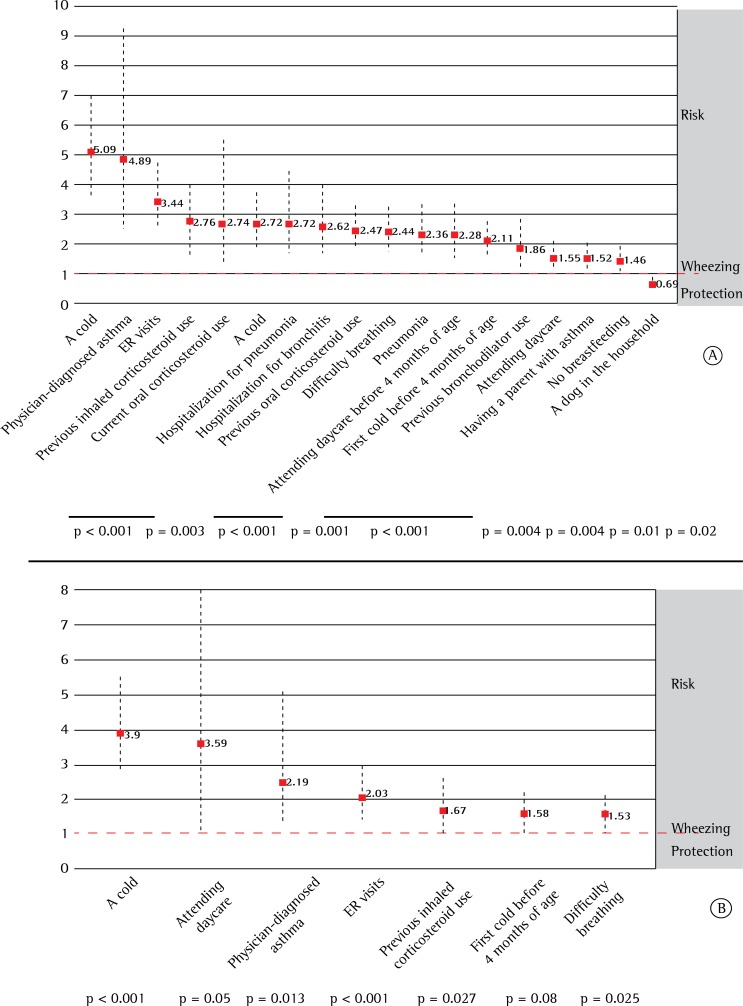
Factors associated with wheezing in the first year of life (wheezing infants, n = 715; nonwheezing infants, n = 554; total, n = 1,269).

The multivariate analysis showed that certain risk factors remained. Figures 2A and 2B show the bivariate and multivariate analyses of risk factors for WBS, respectively. A comparison was made between the OW and WBS groups. Table 2 shows the distribution of risk factors for recurrent wheezing, together with their respective ORs and 95% CIs, among the infants studied. 

**Table 2 -:** Factors associated with recurrent wheezing in the first year of life (occasional wheezing, n = 343; persistent wheezing, n = 232; total, n = 715).

Type of analysis	Variable	OR	95% CI	p
Bivariate	More than three colds in the first year	5.09	3.69-7.01	< 0.001
	Physician-diagnosed asthma	4.89	2.57-9.29	< 0.001
	ER visits	3.44	2.49-4.77	< 0.001
	History of inhaled corticosteroid use	2.76	1.91-3.98	< 0.001
	Current oral corticosteroid use	2.74	1.35-5.54	0.0029
	First cold before 4 months of age	2.72	1.91-3.80	< 0.001
	Hospitalization for pneumonia	2.72	1.64-4.51	< 0.001
	Hospitalization for bronchitis	2.62	1.68-4.07	< 0.001
	History of oral corticosteroid use	2.47	1.81-3.37	0.001
	Difficulty breathing	2.44	1.8-3.31	< 0.001
	Pneumonia	2.36	1.64-3.40	< 0.001
	Attending daycare before 4 months of age	2.28	1.53-3.41	< 0.001
	Age at first cold	2.11	1.56-2.84	< 0.001
	History of bronchodilator use	1.86	1.21-2.86	0.0037
	Attending daycare	1.55	1.14-2.11	0.0044
	Having a relative with asthma	1.52	1.09-2.12	0.0119
	No breastfeeding	1.46	1.09-1.96	0.0108
	Mother working outside the home	1.32	0.95-1.83	0.0933
	A dog in the household	0.69	0.51-0.94	0.0205
Multivariate	More than three colds in the first year	3.90	2.76-5.51	< 0.001
	A cold	3.59	0.98-13.24	0.0545
	Physician-diagnosed asthma	2.49	1.21-5.10	0.013
	ER visits	2.03	1.38-2.97	0.0003
	History of inhaled corticosteroid use	1.67	1.06-2.63	0.0273
	First cold before 4 months of age	1.58	1.13-2.22	0.0082
	Difficulty breathing	1.53	1.06-2.22	0.0246

## Discussion

The present study was the first to determine the prevalence of and risk factors for wheezing in infants in the state of Santa Catarina, Brazil. In comparison with other studies conducted in Brazil and using the EISL questionnaire, ours showed the highest prevalence of wheezing in the first year of life. The prevalence of wheezing in infants was found to be 43% in the city of Recife,^(^ 7 ^)^ 43.2% in the city of Cuiabá,^(^ 8 ^)^ 45.4% in the city of Curitiba,^(^ 3 ^)^ and 46% in the city of São Paulo.^(^ 4 ^)^ The prevalence rates of OW and WBS were as follows: 22,7% and 22.6%, respectively, in Curitiba^(^ 2 ^)^; 19,4% and 26.6%, respectively, in São Paulo^(^ 5 ^)^; 54.1% and 45,9%, respectively, in Cuiabá^(^ 6 ^)^; and 61% and 20%, respectively, in Porto Alegre.^(^ 17 ^)^ In the present study, they were 27,00% and 29.31%, respectively. In Latin America, Europe, and the Netherlands, the prevalence rates of wheezing in infants were found to be 21.4%, 15.0%, and 14.5%, respectively,^(^ 10 ^,^ 11 ^)^ being lower than the prevalence rate found in the present study. 

Social, economic, and environmental characteristics, as well as climate and latitude, have been reported as reasons for the differences in prevalence rates across studies.^(^ 10 ^,^ 16 ^)^

In the present study, the risk factors for OW and WBS were similar to those reported in other studies conducted in Brazil.^(^ 2 ^-^ 7 ^)^ Our study showed an association of WBS with upper and lower airway infections. It is known that infants have many episodes of viral infection,^(^ 18 ^-^ 21 ^)^ and that OW and WBS are associated with many viruses. For most children, episodes of wheezing associated with respiratory infections decrease with age; however, for some, wheezing attacks early in life can mark the beginning of asthma.^(^ 18 ^-^ 21 ^)^

Viral respiratory infections can have serious adverse effects in patients with asthma and account for nearly 80% of all episodes of asthma exacerbation in children and in adults. Whether respiratory infections determine the progression of WBS or the severity of the disease remains unclear. It has been established that some viruses induce asthma, whereas others confer protection against it.^(^ 20 ^)^ Follow-up studies have shown that the timing of birth in relation to the winter virus peak is associated with an increased risk of developing WBS and asthma.^(^ 18 ^-^ 21 ^)^ Such studies suggest that children who are at an increased risk of developing acute viral bronchiolitis are also more likely to develop WBS and asthma.^(^ 17 ^-^ 21 ^)^

In our study, the risk of OW and WBS was found to be higher in the infants who attended daycare than in those who did not. Parents and physicians know that attending daycare in the first two years of life is a risk factor for recurrent respiratory infections. This has been extensively studied. In contrast, conflicting results indicate that the risk of developing asthma is higher^(^ 22 ^,^ 23 ^)^ or lower^(^ 24 ^,^ 25 ^)^ in infants who attend daycare or have older siblings than in those who do not. Therefore, attending daycare as a risk factor for WBS is intriguing because it appears to have contrasting effects. On the one hand, if attending daycare is associated with the presence of recurrent wheezing, the long-term prognosis of such patients is probably good. On the other hand, if attending daycare is a risk factor for severe wheezing, this implies an increased risk of developing asthma. Therefore, long-term studies involving virus isolation are needed in order to clarify the role of attending daycare as a risk factor for persistent wheezing.^(^ 11 ^)^

Our data indicate that male infants are more likely to wheeze than are female infants. This has been verified in other studies, and it is known that, in comparison with girls, boys have narrower airways, greater sensitization to aeroallergens, and higher IgE levels early in life.^(^ 26 ^)^ However, although the prevalence of recurrent wheezing is higher in boys, it decreases as they grow older and reach adolescence.^(^ 1 ^,^ 27 ^)^

Although we did not evaluate the severity of wheezing in the present study, an increasing number of studies have shown the importance of breastfeeding, especially in protecting against severe wheezing episodes in infants. A study evaluating 12,474 children with bronchiolitis, 1,588 of whom required hospitalization, showed that infants whose mothers had not initiated breastfeeding in the maternity ward were at an increased risk of hospitalization for viral bronchiolitis.^(^ 28 ^)^

One group of authors studied a group of infants hospitalized for acute viral bronchiolitis and noted that the duration of breastfeeding was inversely related to the duration of oxygen use and hospitalization, having reported that, for each month of breastfeeding, there was an 11-h reduction in the duration of oxygen use. ^(^ 29 ^)^ Therefore, mothers should be encouraged to prolong breastfeeding and to keep infants under 6 months of age out of daycare. 

Another group of authors^(^ 30 ^)^ found that reduced tobacco exposure and increased intake of oily fish during pregnancy and early childhood can be effective in reducing the incidence of asthma at two years of age. The differential impact on boys and girls suggests that the pathophysiology of asthma depends on the gender of the children. 

One of the risk factors for WBS and asthma in children is a family history of atopy and allergies. In our study, we found that the infants whose parents had asthma and allergies were more likely to have episodes of OW and WBS than were those whose parents had no family history of asthma or allergies. This finding suggests that genetic factors play an important role in OW and WBS. 

One limitation of the present study is that we did not address risk factors that might be specific to the study population. However, this provides an opportunity for studies investigating daycare attendance and the presence/absence of older siblings. 

In conclusion, the prevalence rate of wheezing in infants in the city of Blumenau was 56.34%, of which 27.31% and 29.31% of OW and WBS, respectively. The etiology was multifactorial, and the risk factors were intrinsic and extrinsic, including respiratory tract infections, having a parent with allergies, attending daycare, and early wheezing. The high prevalence of WBS and the intrinsic risk factors indicate the need and the opportunity for epidemiological and genetic studies in this population. 

## References

[1] Martinez FD, Wright AL, Taussig LM, Holberg CJ, Halonen M, Morgan WJ (1995). Asthma and wheezing in the first six years of life. The Group Health Medical Associates. N Engl J Med.

[2] Chong HJ, Rosário NA, Solé D, Mallol J (2007). Prevalence of recurrent wheezing in infants. J Pediat (Rio J).

[3] Chong HJ, Rosário NA, Grupo Curitiba EISL (2008). Risk factors for wheezing in the first year of life. J Pediatr (Rio J).

[4] Dela Bianca AC, Wandalsen GF, Mallol J, Solé D (2010). Prevalence and severity of wheezing in the first year of life. J Bras Pneumol.

[5] Dela Bianca A, Wandalsen G, Mallol J, Sole D (2012). Risk factors for wheezing disorders in infants in the first year of life living in São Paulo, Brazil. J Trop Pediatr.

[6] Moraes LS, Takanoa AO, Mallol J, Solé D (2013). Risk factors associated with wheezing in infants. J Pediatr (Rio J).

[7] Medeiros D, Silva AR, Rizzo JA, Sarinho E, Mallol J, Sole D (2011). Prevalence of wheezing and associated risk factors among infants in Recife, Pernambuco State, Brazil [Article in Portuguese]. Cad Saude Publica.

[8] Rosa AM, Jacobson Lda S, Botelho C, Ignotti E (2013). Prevalence of wheezing and associated factors in children under 5 years of age in Cuiabá, Mato Grosso State, Brazil [Article in Portuguese]. Cad Saude Publica.

[9] Mallol J, Andrade R, Auger F, Rodriguez J, Alvarado R, Figueroa L (2005). Wheezing during the first year of life in infants from low-income population: a descriptive study. Allergol Immunopathol (Madr).

[10] Mallol J, García-Marcos L, Solé D, Brand P, the EISL Study Group (2010). International prevalence of recurrent wheezing during the first year of life: variability, treatment patterns and use of health resources. Thorax.

[11] Visser CA, Garcia-Marcos L, Eggink J, Brand PL (2010). Prevalence and risk factors of wheeze in Dutch infants in their first year of life. Pediatr Pulmonol.

[12] Venero-Fernándes SL, Suárez-Medina R, Mora-Faife EC, García-García G, Valle-Infante I, Gómez-Marrero L (2013). Risk factors for wheezing in infants born in Cuba. QJM.

[13] Guilbert TW, Morgan WJ, Zeiger RS, Bacharier LB, Boehmer SJ, Krawiec M (2004). Atopic characteristics of children with recurrent wheezing at high risk for the development of childhood asthma. J Allergy Clin Immunol.

[14] Mallol J, García-Marcos L, Aguirre V, Martinez-Torres A, Perez-Fernández V, Gallardo A (2007). The International Study of Wheezing in infants: questionnaire validation. Int Arch Allergy Immunol.

[15] Bianca AC, Wandalsen GF, Miyagi K, Camargo L, Cezarin D, Mallol J (2009). International Study of Wheezing in Infants (EISL): validation of written questionnaire for children aged below 3 years. J Investig Allergol Clin Immunol.

[16] Garcia-Marcos L, Mallol J, Solé D, Brand PL, Sanchez-Bahillo M, Sanchez-Solis M (2013). Latitude modifies the effect size of factors related to recurrent wheeze in the first year of life. Respir Med.

[17] Lima JA, Fischer GB, Sarria EE, Mattiello R, Solé D (2010). Prevalence of and risk factors for wheezing in the first year of life. J Bras Pneumol.

[18] Busse WW, Lemanske RF, Gern JE (2010). Role of viral respiratory infections in asthma and asthma exacerbations. Lancet.

[19] Wu P, Dupont WD, Griffin MR, Carroll KN, Mitchel EF, Gebretsadik T (2008). Evidence of a causal role of winter virus infection during infancy in early childhood asthma. Am J Respir Crit Care Med.

[20] Jackson DJ, Gangnon RE, Evans MD, Roberg KA, Anderson EL, Pappas TE (2008). Wheezing rhinovirus illnesses in early life predict asthma development in high-risk children. Am J Respir Crit Care Med.

[21] Kusel MM, de Klerk NH, Kebadze T, Vohma V, Holt PG, Johnston SL (2007). Early-life respiratory viral infections, atopic sensitization, and risk of subsequent development of persistent asthma. J Allergy Clin Immunol.

[22] Sun Y, Sundell J (2011). Early daycare attendance increase the risk for respiratory infections and asthma of children. J Asthma.

[23] Caudri D, Wijga A, Scholtens S, Kerkhof M, Gerritsen J, Ruskamp JM (2009). Early daycare is associated with an increase in airway symptoms in early childhood but is no protection against asthma or atopy at 8 years. Am J Respir Crit Care Med.

[24] Ball TM, Castro-Rodriguez JA, Griffith KA, Holberg CJ, Martinez FD, Wright AL (2000). Siblings, day-care attendance, and the risk of asthma and wheezing during childhood. N Engl J Med.

[25] Gaffin JM, Spergel JM, Boguniewicz M, Eichenfield LF, Paller AS, Fowler JF (2012). Effect of cat and daycare exposures on the risk of asthma in children with atopic dermatitis. Allergy Asthma Proc.

[26] Sherrill DL, Stein R, Halonen M, Holberg CJ, Wright A, Martinez FD (1999). Total serum IgE and its association with asthma symptoms and allergic sensitization among children. J Allergy Clin Immunol.

[27] van Merode T, Maas T, Twellaar M, Kester A, van Schayck CP (2007). Gender-specific differences in the prevention of asthma-like symptoms in high-risk infants. Pediatr Allergy Immunol.

[28] Koehoorn M, Karr CJ, Demers PA, Lencar C, Tamburic L, Brauer M (2008). Descriptive epidemiological features of bronchiolitis in a population-based cohort. Pediatrics.

[29] Dornelles CT, Piva JP, Marostica PJ (2007). Nutritional status, breastfeeding, and evolution of Infants with acute viral bronchiolitis. J Health Popul Nutr.

[30] Dotterud CK, Storrø O, Simpson MR, Johnsen R, Øien T (2013). The impact of pre- and postnatal exposures on allergy related diseases in childhood: a controlled multicentre intervention study in primary health care. BMC Public Health.

